# Lack of correlation between hippocampal substructure atrophy and attention dysfunction in deficit schizophrenia

**DOI:** 10.1038/s41537-023-00354-z

**Published:** 2023-04-20

**Authors:** Jin Li, Xiaobin Zhang, Haidong Yang, Man Yang, Hongyan Sun

**Affiliations:** 1 Institute of Mental Health, Suzhou Psychiatric Hospital, The Affiliated Guangji Hospital of Soochow University, 11 Guangqian Road, Suzhou, 215137 Jiangsu China; 2 Department of Psychiatry, The Fourth People’s Hospital of Lianyungang, The Affiliated KangDa College of Nanjing Medical University, Lianyungang, 222003 PR China

**Keywords:** Schizophrenia, Working memory

## Abstract

Hippocampal abnormalities are an established finding in the neuroimaging study of schizophrenia. However, no studies have examined the possibility of regional hippocampal abnormalities specific to deficit schizophrenia (DS) and associations with the unique symptoms of this schizophrenia subtype. This study compared 33 DS and 39 non-deficit schizophrenia (NDS) patients and 38 healthy subjects for hippocampal subfield volumetry. Clinical symptoms were assessed by PANSS, cognition by the neurocognitive battery on the day of the MRI scan. The automatic hippocampal segmentation were preprocesses use FreeSurfer 7.2.0. Unfortunately, the associations between neurocognitive scores and hippocampal subfield volumes in the DS group were not significant after the Bonferroni correction. Our results did not support a causal relationship between hippocampal subregional atrophy and cognitive deficits in DS.

## Introduction

Deficit schizophrenia (DS) is considered a distinct disease entity within the schizophrenia spectrum due to unique clinical manifestations compared to other entities (collectively termed non-deficit schizophrenia or NDS). Notably, DS is characterized by predominant negative symptoms from onset and during clinical stabilization^1^. Many studies have provided evidence for the long-term stability of defect syndrome and the reliability of deficit/non-deficit classification in schizophrenia^2^. There is also evidence that DS can be differentiated from NDS by greater cognitive impairment, poorer treatment response, and worse prognosis^3^. Several studies have reported that patients with DS perform worse on cognitive domains associated with negative symptoms^4^, including executive function^5^, cognitive flexibility^6^, and sustained attention^7^. However, a recent meta-analysis found that DS patients performed at lower levels than NDS patients in all major cognitive domains, and that deficit syndrome severity was strongly associated with cognitive impairment^4^. These neuropsychological and clinicopathological findings suggest that deficit schizophrenia may be a distinct disorder with unique underlying brain pathologies^8,9^.

It is well established that the hippocampus is essential for various forms of declarative (explicit) memory^10^, including verbal memory^11^. Numerous studies conducted over many decades have consistently found that hippocampal damage or atrophy impairs performance on declarative memory tasks, including among patients with schizophrenia^12–15^. The hippocampus is thought to play a crucial role in mediating attention^16^, navigating physical space^17^, semantic fluency^18^ and executive function^19^. A recent meta-analysis concluded that hippocampal volume is reduced even at disease onset as well as during the chronic stage^13^, and studies of first-episode patients have suggested that this early atrophy contributes to pathogenesis^20,21^. The left hippocampal volume was reduced in individuals in a clinically high-risk psychotic state^22^. The first-degree relatives of schizophrenic patients had smaller hippocampal volumes than healthy control subjects^23^. A recent multicenter neuroimaging study also found that the most obvious structural anomalies in patients with schizophrenia were within the hippocampus^24^. However, no studies have examined the possibility of regional hippocampal abnormalities specific to DS and associations with the unique symptoms of this schizophrenia subtype.

The hippocampus consists of distinct structurally and functionally distinct regions and subregions, including cornu ammonis (CA) fields 1 to 4 (CA1–CA4), the dentate gyrus (DG), and subiculum^12–14^. A recent systematic review of hippocampal neuroimaging studies concluded that the volumes of these hippocampal regions and subregions are differentially affected in schizophrenia, with CA1 most severely altered^25^. Schobel et al reported hypermetabolism in CA1 leading to atrophy in the early stages of schizophrenia^26^ and further studies on serotonin modulation of hippocampal functions suggested that the onset of schizophrenia symptoms is associated with hippocampal excitotoxicity triggered by stress-induced hyperactivity of serotonergic pacemaker cells in the dorsal raphe nucleus^27,28^. Consistent with distinct effects on hippocampal subregions during the disease course, Ho and colleagues found progressive disease-related volume loss extending initially from CA1 to all other subregions^11^. Hippocampal substructure volume atrophy has also been confirmed in first-episode drug-naive psychotic patients; moreover, the same study found a non-linear relationship between dentate gyrus/CA4 volume changes and antipsychotic dose after 12 weeks of risperidone or aripiprazole treatment^29^. Further, more recent studies have found significant associations of cognitive composite and declarative memory scores with the volumes of multiple hippocampal substructures in schizophrenia^25,30^.

Based on these findings, we hypothesized that subfield analysis would yield better predictors of symptom progression in DS compared to total hippocampal volume, and that DS patients would present with greater regional volume reductions compared to NDS patients, leading to more severe cognitive impairment.

## Materials and methods

### Participants

This cross-sectional comparative study included 72 patients with schizophrenia (33 DS and 39 NDS patients) and 38 healthy subjects from the Department of Psychiatric Rehabilitation, Wutaishan Hospital, Yangzhou City, Jiangsu Province, China. All patients were inpatients and right-handed males between 20 and 65 years of age diagnosed according to the Diagnostic and Statistical Manual of Mental Disorders Fourth Edition (DSM-IV) criteria and the DSM-IV Structured Clinical Interview (SCID-I)^31^. Each patient was on antipsychotic medications and the regimen had not changed for at least 12 months prior to the study. In addition to study candidates with recent changes in medication, we excluded candidates with neurological diseases, previous head trauma, mental retardation, past or current history of alcohol or illicit drug abuse/dependence, and history of electroconvulsive therapy. Schizophrenic patients who meet the above criteria were jointly assessed as DS or NDS by two attending physicians using the Chinese version of the Deficiency Syndrome Scale (SDS)^32^. The diagnostic criteria for deficit schizophrenia are: 1. Symptomatologic criteria: six negative symptom clusters are included: (1) restricted affect, (2) diminished emotional range, (3) poverty of speech, (4) curbing of interests, (5) diminished sense of purpose, and (6) diminished social drive. The above symptom clusters can be grouped into two factors^33,34^: the affective expression factor (1-3) and the lack of motivation factor (4-6); 2. Severity and course criteria: two or more of the above negative symptoms have been clinically significant and have persisted for more than 12 months, and also persist during the clinical stabilization period; 3. The above symptoms are primary or idiopathic, not secondary to depression, anxiety, drug side effects, psychotic symptoms, or mental retardation; 4. The DSM-IV diagnosis of schizophrenia is met. Where each negative symptom cluster is scored on a 5-point scale of 0-4 (0: normal; 1: possible problem, but no significant abnormality, or there are some unusual manifestations, but they are still within the normal range of variation; 2 or 3 points: clinically, the symptom group of the patient is obviously abnormal; 4 points: the patient’s symptom group is extremely serious). Clinical significance is defined as the score of the symptom group is greater than or equal to 2 points. Those that do not meet this standard are NDS.

Healthy controls were matched for age, handedness, and education, and candidates with a history of cognitive, psychiatric, or physical co-morbidities were excluded.

The study was approved by the Institutional Ethics Committee for clinical research (approval No. 2018-056) and all participants provided written informed consent.

### Clinical assessments

Clinical symptoms were assessed by a trained research psychiatrist using the Positive and Negative Syndrome Scale (PANSS) on the day of the MRI scan.

### Neurocognitive assessments

We conducted the neurocognitive battery on all subjects, including the Digit Cancellation Test (DCT), Animal Naming Test (ANT), Controlled Oral Word Association Test (COWAT), Spatial Span Test (SS), two-part Trail Making Task (TMT-A and TMT-B), Block Design Test (BDT) and the Stroop Color-word Test (SCWT). All participants performed practice tests to ensure that they understood the instructions of the examiner.

### MRI acquisition

All magnetic resonance images were acquired on a 3.0-T MRI scanner (GE HDx) using a T1-weighted high-resolution three-dimensional brain volume imaging (3D-BRAVO) sequence with the following parameters: TR = 11.94 ms; TE = 5.044 ms; flip angle = 15°; field of view = 240 × 240 mm; matrix size = 256 × 256; slice thickness = 1 mm without gaps; voxel size = 1 × 1 × 1 mm3; number of slices = 172. During the scans, participants were instructed to remain awake with their eyes closed, to lie still, and not to focus on anything specific. All reconstructed images are visually inspected by trained researchers, and the images filtered through visual inspection will enter the FreeSurfer processing.

### Imaging data preprocessing

All images were preprocesses use FreeSurfer 7.2.0, which is available for free online download (http://surfer.nmr.mgh.harvard.edu/).The hippocampus was automatically segmented into 12 distinct regions: hippocampal tail, subiculum, CA1, hippocampal fissure, presubiculum, parasubiculum, molecular layer hippocampus (HP), granule cell and molecular layer of the dentate gyrus (GC-ML-DG), CA3, CA4, fimbria, and hippocampus-amygdala-transition-area (HATA). The partitioning algorithm is based on a computational atlas constructed using the ex vivo MRI data of the medial temporal lobe from cadaveric brains. The results of the program run have no error.

### Statistical analysis

All statistical analyses were performed using SPSS 17.0 (SPSS Inc., Chicago, IL, USA). Participant demographics, clinical characteristics, and neurocognitive assessment scores are presented as mean ± standard deviation (SD). Means of demographic and clinical characteristics were compared among DS, NDS, and HC groups by one-way analysis of variance (ANOVA) and post-hoc pairwise comparison analyses, while differences in psychiatric symptoms were compared between DS and NDS groups by independent sample *t* tests. Hippocampal subregion volumes were compared among groups using analysis of covariance (ANCOVA) controlling for age, education, eTIV (estimated Total Intracranial Volume), and body mass index (BMI), and adjusted for multiple comparisons using the Bonferroni correction. Cognition except COWAT scores were compared among groups by ANCOVA controlling for age, education, eTIV, and BMI, with adjustment for multiple comparisons using Bonferroni correction, while COWAT results were compared by one-way ANOVA with Bonferroni correction. Datasets were first examined for homogeneity of variance using Welch’s test. A separate multiple linear regression model was constructed to investigate whether hippocampal subregion volumes were associated with cognitive tests scores. The *P*-value was set to 0.05. Bonferroni correction was applied to adjust for multiple comparisons. The Bonferroni-corrected *P* value for each PANSS score is the initial *P* value ×4. The Bonferroni-corrected *P* value for each hippocampal subfield volume is the initial *P* value ×44. The Bonferroni-corrected *P* value for each cognitive test score is the initial *P* value ×11. We will not do Bonferroni correction when the initial *P* value >0.05.

## Results

### Demographic data in patients and controls

The demographic and clinical characteristics of all 110 participants are summarized in Table 1. There were significant differences in years of education and BMI among DS, NDS, and HC groups, and post hoc analysis indicated that years of formal education was lowest in the DS group and that BMI was lowest in the HC group. Age at onset and CPZ equivalent dose did not differ significantly between the DS and NDS groups, but disease duration was significantly longer in the DS group. Total PANSS score was significantly higher in the DS group than the NDS group, indicating more severe clinical symptoms. In addition, negative syndrome and general psychopathological syndrome subscores of the PANSS were significantly higher in the DS group than the NDS group, while positive syndrome subscores did not differ between these groups.

**Table 1:** Demographics and clinical characteristics of DS, NDS, and HC groups.

	DS (*n* = 33)	NDS (*n* = 39)	HC (*n* = 38)	F/t	*P* value	Bonferroni correction *P* value
Age	49.12 ± 7.71	45.92 ± 5.47	46.13 ± 9.11	1.946	0.147836	–
Education years	8.15 ± 2.96	9.18 ± 1.85	10.50 ± 2.83	7.487	0.000905^*^	–
BMI	24.06 ± 3.45	25.66 ± 2.96	23.66 ± 2.16	5.230	0.006820^*^	–
eTIV	1537675.79 ± 127331.11	1530514.84 ± 279059.61	1453646.92 ± 124356.50	2.123	0.124722	–
Age at onset	22.12 ± 2.72	22.51 ± 2.66	–	−0.615	0.540517	–
Duration	27.00 ± 6.93	23.41 ± 5.95	–	2.366	0.020736^*^	–
CPZ-equivalent dose(mg/day)	486.52 ± 225.20	545.64 ± 204.80	–	−1.166	0.247521	–
PANSS total score	68.33 ± 3.76	50.03 ± 3.38	–	21.728	7.8019E−33	0.000^*^
Positive syndrome	11.03 ± 1.05	10.72 ± 2.05	–	0.832	0.408905	–
Negative syndrome	26.70 ± 2.33	14.26 ± 1.35	–	27.106	2.3168E−31	0.000^*^
General psychopathology syndrome	30.58 ± 2.83	25.05 ± 2.19	–	9.338	6.3699E−14	0.000^*^

Values presented as mean ± standard deviation (SD).

*DS* deficit schizophrenia, *NDS* non-deficit schizophrenia, *HC* healthy controls, *PANSS* Positive and Negative Syndrome Scale, *CPZ* chlorpromazine, *BMI* body mass index, *eTIV* estimated Total Intracranial Volume, *E* exponent (Scientific Notation).

*P < 0.05.

### Hippocampal subfield volumetry

The volumes of 8 subregions in the left hippocampus (left hippocampal GC-ML-DG body, left hippocampal CA1 head, left hippocampal molecular layer head, left hippocampal molecular layer body, left hippocampal fimbria, left hippocampal head, left hippocampal body, left hippocampus) and 12 subregions in the right hippocampus (right hippocampal GC-ML-DG head, right hippocampal GC-ML-DG body, right hippocampal CA4 head, right hippocampal CA4 body, right hippocampal CA1 head, right hippocampal CA1 body, right hippocampal molecular layer head, right hippocampal molecular layer body,

right hippocampal fimbria, right hippocampal head, right hippocampal body, right hippocampus) were significantly reduced in schizophrenia patients (combined DS and NDS group) compared to HCs (all *P* < 05 by ANCOVA controlling for age, education, eTIV and BMI, and with Bonferroni correction for multiple comparisons). The post-hoc-comparisons found that the volumes of hippocampal substructure in DS and NDS groups were significantly smaller than that in the normal control group, but there was no significant difference between DS and NDS groups (Table 2).

**Table 2:** Hippocampal subfield volumes of DS, NDS, and HC groups.

	Hippocampal substructure	DS (*n* = 33)	NDS (*n* = 39)	HC (*n* = 38)	F	*P* value	Bonferroni correction *P* value
Left hippocampal	GC-ML-DG head	145.52 ± 22.06	146.47 ± 19.60	163.18 ± 19.66	6.680	0.001878	0.083
	GC-ML-DG body	137.03 ± 22.34	138.62 ± 15.98	154.67 ± 12.92	10.148	0.00096	0.004*^a^
	CA4 head	122.81 ± 17.18	122.37 ± 15.81	135.53 ± 16.74	5.873	0.003854	0.169
	CA4 body	122.54 ± 18.42	125.03 ± 15.03	136.61 ± 12.14	7.448	0.000957	0.042
	CA3 head	117.18 ± 21.16	116.77 ± 18.53	127.14 ± 17.83	2.594	0.079674	–
	CA3 body	90.71 ± 18.18	93.62 ± 113.99	100.16 ± 15.64	2.848	0.062561	–
	CA1 head	503.37 ± 61.89	496.96 ± 52.25	556.78 ± 57.99	9.786	0.000129	0.008^*a^
	CA1 body	117.47 ± 21.8	117.44 ± 23.63	130.57 ± 21.08	2.558	0.082450	–
	Subiculum head	171.57 ± 20.78	177.22 ± 28.47	191.47 ± 26.32	6.407	0.002392	0.105
	Subiculum body	252.32 ± 34.12	265.61 ± 31.94	281.49 ± 24.32	5.636	0.004767	0.210
	Presubiculum head	129.73 ± 19.41	131.76 ± 13.54	141.10 ± 11.97	6.552	0.002103	0.093
	Presubiculum body	178.45 ± 30.81	186.51 ± 28.09	193.61 ± 26.10	2.999	0.054252	–
	Parasubiculum	60.69 ± 19.93	59.17 ± 12.88	65.08 ± 13.79	1.676	0.192208	–
	Molecular layer head	315.96 ± 38.72	317.11 ± 31.39	351.83 ± 33.56	11.616	0.000029	0.001*^a^
	Molecular layer body	219.97 ± 34.69	226.37 ± 26.97	249.79 ± 24.41	8.463	0.000398	0.018*^a^
	HATA	56.25 ± 11.65	57.16 ± 8.53	59.45 ± 7.80	1.741	0.180503	–
	Tail	575.35 ± 96.11	571.7068.82	622.96 ± 85.92	2.803	0.065295	–
	Fissure	148.63 ± 28.73	145.59 ± 25.27	137.41 ± 22.08	0.495	0.611302	–
	Fimbria	60.04 ± 27.02	66.21 ± 22.10	82.28 ± 18.16	7.727	0.000750	0.033*^a^
	Head	1623.07 ± 201.50	1624.98 ± 158.73	1791.57 ± 162.49	10.157	0.000095	0.004*^a^
	Body	1178.52 ± 169.65	1219.09 ± 117.54	1329.18 ± 98.03	10.747	0.000058	0.003*^a^
Left hippocampus		3376.94 ± 404.13	3415.77 ± 271.60	3743.71 ± 283.33	12.171	0.000018	0.001*^a^
Right hippocampal	GC-ML-DG head	149.92 ± 318.84	153.28 ± 19.57	173.59 ± 21.90	10.786	0.000056	0.002*^a^
	GC-ML-DG body	141.92 ± 22.38	143.5813.74	160.81 ± 17.30	10.368	0.000080	0.004*^a^
	CA4 head	127.44 ± 113.89	128.41 ± 115.32	143.94 ± 17.91	9.682	0.000141	0.006*^a^
	CA4 body	127.94 ± 19.89	129.46 ± 12.95	142.84 ± 14.85	8.212	0.000494	0.022*^a^
	CA3 head	121.71 ± 16.54	121.66 ± 19.31	138.49 ± 19.40	7.185	0.001204	0.053
	CA3 body	100.22 ± 23.40	104.84 ± 15.54	115.47 ± 14.64	4.893	0.009349	0.411
	CA1 head	516.39 ± 65.45	527.91 ± 58.17	595.09 ± 73.21	11.515	0.000031	0.001*^a^
	CA1 body	124.57 ± 29.00	128.22 ± 21.52	150.08 ± 29.20	8.271	0.000469	0.021*^a^
	Subiculum head	180.11 ± 29.56	185.57 ± 23.68	199.54 ± 29.38	3.876	0.023850	1.049
	Subiculum body	253.58 ± 39.15	257.19 ± 20.97	278.81 ± 39.59	5.716	0.004437	0.195
	Presubiculum head	125.05 ± 20.08	129.30 ± 14.10	140.20 ± 18.01	5.414	0.005824	0.256
	Presubiculum body	167.56 ± 29.69	170.71 ± 25.17	180.08 ± 38.15	1.323	0.270860	–
	Parasubiculum	56.99 ± 18.22	51.62 ± 10.66	57.59 ± 13.95	2.647	0.075748	–
	Molecular layer head	324.06 ± 40.97	332.42 ± 34.70	370.18 ± 41.58	11.098	0.000044	0.002*^a^
	Molecular layer body	228.62 ± 39.27	236.28 ± 23.00	268.72 ± 35.36	11.838	0.000024	0.001*^a^
	HATA	53.92 ± 12.01	54.09 ± 8.34	61.13 ± 9.41	5.560	0.005106	0.225
	Tail	604.78 ± 85.36	604.26 ± 69.84	667.91 ± 75.89	6.122	0.003084	0.136
	Fissure	161.76 ± 33.67	155.75 ± 25.91	143.83 ± 26.54	1.435	0.242961	–
	Fimbria	59.72 ± 26.11	67.86 ± 19.89	83.54 ± 16.70	8.485	0.000390	0.017*^a^
	Head	1655.60 ± 195.53	1684.27 ± 175.61	1879.75 ± 214.12	11.171	0.000041	0.002*^a^
	Body	1204.13 ± 183.61	1238.13 ± 94.96	1380.35 ± 171.26	11.395	0.000034	0.001*^a^
Right hippocampus		3464.51 ± 419.68	3526.66 ± 291.26	3928.01 ± 393.64	14.016	0.000004	0.002*^a^

Values presented as mean ± S.D.

*DS* deficit schizophrenia, *NDS* non-deficit schizophrenia, *HC* healthy control, *CA* cornu ammonis, *GC-ML-DG* granule cell layer and molecular layer of the dentate gyrus, *HATA* hippocampal amygdala transition area.

Results compared by analysis of covariance (ANCOVA) controlling for age, education, eTIV and BMI, and adjusted for multiple comparisons using Bonferroni correction; ^*^P < 0.05; ^a^ The post-hoc-comparisons found that the volumes of hippocampal substructure in DS and NDS groups were significantly smaller than that in normal control group, but there was no significant difference between DS and NDS groups.

### Neuropsychological assessment

Schizophrenia patients performed significantly worse than HCs on all cognitive tests, and the DS group performed significantly worse than the NDS group (Table 3).

**Table 3:** Cognitive test scores of DS, NDS, and HC groups.

	DS (*n* = 33)	NDS (*n* = 39)	HC (*n* = 38)	F/Asymptotically F distributed	*P* value	Bonferroni correction *P* value
DCT^a^	333.17 ± 248.50	182.78 ± 66.06	139.46 ± 43.38	14.191	0.000010	0.000^b^
ANT	9.30 ± 3.33	12.21 ± 4.54	18.50 ± 4.62	31.979	3.4428E−12	0.000^c^
COWAT^d^	4.67 ± 3.18	6.74 ± 3.57	8.97 ± 2.32	21.395	6.3928E−8	0.000^e^
Category fluency score	13.97 ± 5.58	18.95 ± 6.87	27.47 ± 5.52	31.030	4.0133E−12	0.000^e^
Spatial span total	11.12 ± 4.44	13.10 ± 3.52	18.05 ± 3.52	17.541	3.8095E−8	0.000^c^
TMT-A(s)^a^	144.13 ± 73.84	81.67 ± 31.43	50.25 ± 23.87	31.246	4.3971 E−10	0.000^e^
TMT-B(s)	316.59 ± 124.55	198.57 ± 54.54	124.24 ± 66.04	33.621	3.4359E−12	0.000^e^
Block design	13.48 ± 8.72	21.44 ± 6.62	27.97 ± 8.42	19.594	3.0497E−8	0.000^e^
SCWT						
Stroop-words	42.64 ± 18.73	58.51 ± 15.38	79.47 ± 16.36	27.172	3.6198E−11	0.000^e^
Stroop-colors	25.64 ± 12.85	35.59 ± 11.35	49.42 ± 13.46	19.296	2.1756E−8	0.000^e^
Stroop-interference	15.97 ± 10.84	21.41 ± 8.78	32.50 ± 10.76	15.820	4.2205E−7	0.000^c^

Values presented as mean ± S.D.

*DS* deficit schizophrenia, *NDS* non-deficit schizophrenia, *HC* healthy control, *DCT* Digit Cancellation Test, *ANT* Animal Naming Test, *COWAT* Controlled Oral Word Association Test, *TMT* Trail-Making Test, *SCWT* Stroop Color-word Test, *E* exponent (Scientific Notation).

^a^Not meeting homogeneity of variances using Welch’s test. All other cognitive test scores were compared by analysis of covariance (ANCOVA) controlling for age, education and BMI. All statistical test results were adjusted for multiple comparisons using Bonferroni correction.

^b^The post-hoc-comparisons found that the scores of DCT in DS groups were significantly lower than that in HC and NDS groups, but there was no significant difference between HC and NDS groups.

^c^The post-hoc-comparisons found that the scores of neurocognitive battery in DS and NDS groups were significantly lower than that in HC group, but there was no significant difference between DS and NDS groups.

^d^Education could not be excluded as a confounding factor, so one-way analysis of variance (ANOVA) was used to compare scores among groups.

^e^The post-hoc-comparisons found that the scores of neurocognitive battery in DS and NDS groups were significantly lower than that in HC group, the scores of neurocognitive battery in DS were significantly lower than that in NDS group.

### Associations of neurocognitive scores and hippocampal subfields volumes

In the DS group, multiple regression analysis controlling for age, education, and BMI revealed that smaller right hippocampal CA1 head volume (*p* = 0.004) was significantly associated with worse DCT performance, while smaller left hippocampal tail volume (*p* = 0.003) and smaller right hippocampal HATA volume (*p* = 0.021) were associated with worse category fluency scores. In addition, smaller right hippocampal molecular layer head volume was associated with worse Stroop-colors task performance (*p* = 0.016). Unfortunately, none of the above correlations were significant after the Bonferroni correction. In the HC group, there were no significant associations after Bonferroni correction.

## Discussion

Morphometric analysis of structural magnetic resonance images revealed significantly reduced CA1, CA4, molecular layer, fimbria, and GC-ML-DG volumes in chronic schizophrenia patients compared to matched healthy controls. Unfortunately, in DS patients, atrophy of hippocampal subfield volumes was not associated with neurocognitive scores after the Bonferroni correction. Our results did not support a causal relationship between hippocampal subregional atrophy and cognitive deficits in DS.

This study identifies several regions of hippocampal atrophy that could serve as biomarkers of cognitive dysfunction in schizophrenia and as targets for future investigations on disease pathogenesis. Decreased hippocampal volume is a consistent finding in neuroimaging studies of patients with schizophrenia, although the cellular bases for these volume changes are still unclear. A meta-analysis of autopsy studies found that the volumes of multiple left hippocampal subregions were significantly reduced in schizophrenia patients and that these volume decreases were associated with reduced neuronal size but not density^35^, while no changes were found in the right hippocampus, in contrast to the present study. A study of individuals in a clinically high-risk psychotic state eventually progressing to schizophrenia suggested that left hippocampal volume is reduced before onset and continues to shrink thereafter^22^. Consistent with our findings, Buchanan et al.^36^ found that both schizophrenic subgroups had smaller hippocampal volumes than the normal subjects and there were no differences between the two schizophrenic subgroups in hippocampal volumes. Although there is strong evidence that regional hippocampal atrophy is associated with cognitive impairment in schizophrenics^37^, it is regrettable that we have not found a relationship between regional hippocampal atrophy and specific cognitive impairment in DS patients. The volumes of hippocampal substructure in the DS and NDS groups were significantly smaller than that in the normal control group. The volume of hippocampal substructure in the DS group showed a trend of decrease compared with that in the NDS group (0.06-0.08), but it did not reach statistical significance. The illness duration may significantly impact the magnitude of hippocampal volume deficits in chronic schizophrenia^38,39^. we will conduct a follow-up and longitudinal study to explore the effect of the disease course on the pathological changes of the hippocampus in DS.

Performance on all cognitive domains was significantly lower in schizophrenia patients than healthy controls, and the performance of DS patients was significantly worse than that of NDS patients. These findings are consistent with previous studies showing that DS patients performed worse than NDS patients in every neuropsychological measure and cognitive domain examined^7,40^. The evidence presented in this study supports the hypothesis that DS may be a distinct disease entity within the schizophrenia spectrum. It is still unclear, however, whether these cognitive deficits are pervasive or more severe within certain specific domains. In this regard, one recent study utilizing eye movement analysis suggested that the major deficits were related to attention^41^, while another suggested that DS patients had significantly impaired overall cognitive abilities, possibly associated with chronic neuroinflammation^42^. While the true extent of cognitive deficits in DS remains uncertain, the current study supports more pervasive deficits, at least in patients with a long (several decade) disease history.

This article is the first to report associations between regional hippocampal atrophy and specific cognitive impairments in DS patients. We investigated multiple cognitive functions including attention and executive functions. But it is well known that the hippocampus is essential for human declarative memory^10^. The hippocampus is involved in memory encoding and regulation of emotional behavior among other high-order behavioral processes^43^. Information about new experiences, stimulus exposure, and various associations is thought to be processed in the hippocampus and stored elsewhere in the brain, enabling people to consciously recall personal events (episodic memory) and facts (semantic memory)^43^. The major input–out neurons of the hippocampus are glutamatergic pyramidal cells that span all four CA subregions (CA1, CA2, CA3, CA4) and have distinct input and projection patterns within and outside the hippocampal formation^44^. The key input pathway is called the perforant pathway from the entorhinal cortex^45^, which transmits signals from other brain regions, including the neocortex, to various neuronal populations in the subiculum, CA1, CA3, and dentate gyrus^46^, while output fibers leave the hippocampus via the fornix through the alveus and fimbria^47^.

In principle, any or all of these circuits could be disrupted in schizophrenia, possibly through altered connectivity or deficient synaptic plasticity. Alternatively, global regulation of these circuits by dopaminergic and serotonergic inputs could be dysfunctional. We found that more pronounced CA1 atrophy was associated with worse performance on the DCT in the DS group, while there were no significant correlations between CA1 volume and cognitive function in the NDS or HC groups. In accord with these results, Alden and colleagues reported a correlation between hippocampal deformation and verbal working memory impairments in patients with schizophrenia^48^ while other have reported a correlation between poorer executive function and CA1 volume atrophy in DS patients^40,42^. The DCT assesses focused, sustained, and selective attention^49^, information processing speed, and executive function. Studies have shown that as an attention assessment tool, DCT is associated with prefrontal cognitive function^50,51^. Our findings suggest that CA1 atrophy in DS patients may disrupt the circuit between the hippocampus and frontal cortex.

Findings could be reinforced by larger samples, especially increasing the size of DS patients. The disadvantage of automatic hippocampal segmentation is the concern about the accuracy of mapping subfield boundaries; the advantage is that it is unbiased, less labor-intensive, and easier to reproduce. In addition, there is considerable heterogeneity in the different antipsychotic drugs used by patients. Subgroup and multivariate analyses of sufficient size are needed to hedge against these limitations. Growing preclinical^52^ and clinical^53^ evidence suggests that hippocampal pathology in schizophrenia is localized to the anterior hippocampus. The anterior/posterior gradient of the hippocampus is not discerned. This study attempts to restrict variance due to confounders including gender, illness duration, fluctuation of mental symptoms, and social environment. Future research should also consider out-patients, women, and longitudinal data. Because automatic segmentation of the hippocampus of schizophrenic patients using FreeSurfer may fail^54^, future research should include manual correction of automatic segmentation in case of failure. Some studies have found that cariprazine^55^ and roluperidone^56^ can treat negative symptoms. We will try to intervene in negative symptoms in future studies.

## Conclussion

To date, few studies have investigated the pattern of hippocampal atrophy at the subregional level in DS patients. We found that the cognitive function of the HC, NDS, DS groups showed a horizontal step-wise decline, accompanied by a subregional level of hippocampal atrophy. This subregion-level volume shrinkage was associated with poor performance on tests of cognitive function. Studying hippocampal subregional atrophy patterns has important implications for gaining a deeper understanding of the pathophysiology of cognitive deficits in DS. An immediate clinical implication is the identification of subregional atrophy patterns in DS, thereby enabling clinicians to identify patients with first-episode schizophrenia who are at higher risk for cognitive impairment.

## Supplementary information

the description of the cognitive tasks

## Data Availability

The data that support the findings of this study are available from the corresponding author, upon reasonable request.
