# Supplementary material for: Lack of correlation between hippocampal substructure atrophy and attention dysfunction in deficit schizophrenia
Source: Schizophrenia (Heidelb). 2023 Apr 20;9(1):24. doi: 10.1038/s41537-023-00354-z (PMC10119300; doi:10.1038/s41537-023-00354-z)
Supplement: Supplementary file 1 — the description of the cognitive tasks [file 41537_2023_354_MOESM1_ESM.docx]

For assessment of focused, sustained, and selective attention, all participants performed the Digit Cancellation Test (DCT), in which the subject is asked to delete all numbers in each row that are identical to the first number as quickly and accurately as possible by marking with a slash^1^. To measure semantic language fluency and executive function, participants performed the Animal Naming Test (ANT) and the Controlled Oral Word Association Test (COWAT) in which individuals must provide as many examples as possible from a given category, such as animals, within a preset time of 60 seconds^2^. The sum of ANT and COWAT scores is considered a measure of category fluency. To assess spatial memory, participants performed the Spatial Span Test (SS), in which subjects are asked to repeat the touch pattern of the examiner on a grid of blue squares^3^. To measure processing speed and mental flexibility, participants performed the two-part Trail Making Task (TMT-A and TMT-B), which requires the individual to connect 25 randomly spaced circled numbers in order (1→2→3…etc.) as quickly as possible (TMT-A) or randomly spaced circled numbers and letters in numeric and alphabetical order (e.g., 1→A→2→B...etc.) ^4^. Visuospatial construction capacity was measured using the Block Design Test (BDT), which requires the subject to arrange a set of 4 or 9 two-color blocks to match 10 target patterns in ascending order of difficulty^5^. Response inhibition and selective attention were assess using the Stroop Color-word Test (SCWT), which included Stroop colors, Stroop words, and Stroop distractions^6^. A typical Stroop task requires subjects to correctly read a color name (e.g., ‘RED’ or ‘GREEN’) written in a different colored font (e.g., ‘RED’ in green font). The neurocognitive battery was administered in a quiet room by a trained examiner on the same day as the MRI scan, and all tests were performed in the same order. Patients had not taken antipsychotic medication within the past 2 hours of the test session.

# References

1. Takeshi H, Kazuhito Y, Yasuhiro I, Mitsuhito M, Hidehiro K. RELIABILITY AND VALIDITY OF THE DIGIT CANCELLATION TEST, A BRIEF SCREEN OF ATTENTION. *PSYCHOLOGIA* **55**, 246-256 (2013).

2. Chen YL, Chen YH, Lieh-Mak F. Semantic verbal fluency deficit as a familial trait marker in schizophrenia. *Psychiatry Res* **95**, 133-148 (2000).

3. Woods DL, Wyma JM, Herron TJ, Yund EW. An improved spatial span test of visuospatial memory. *Memory* **24**, 1142-1155 (2016).

4. Reitan RM. Trail Making Test: Manual for Administration and Scoring. *neuropsychological interpretation of objective psychological tests*, (1992).

5. Yin S, Zhu X, Huang X, Li J. Visuospatial characteristics of an elderly Chinese population: results from the WAIS-R block design test. *Front Aging Neurosci* **7**, 17 (2015).

6. Niitsu T*, et al.* Associations of serum brain-derived neurotrophic factor with cognitive impairments and negative symptoms in schizophrenia. *Prog Neuropsychopharmacol Biol Psychiatry* **35**, 1836-1840 (2011).
